# A Review of the Utility of Established Cell Lines for Isolation and Propagation of the Southern African Territories Serotypes of Foot-and-Mouth Disease Virus

**DOI:** 10.3390/v17010039

**Published:** 2024-12-30

**Authors:** Kitsiso Gaboiphiwe, Tshephang Iris Kabelo, Petronella Thato Mosholombe, Joseph Hyera, Elliot Mpolokang Fana, Kabo Masisi, Kebaneilwe Lebani

**Affiliations:** 1 World Organisation for Animal Health (WOAH) Foot-and-Mouth Disease Reference Laboratory, Botswana Vaccine Institute, Private Bag 0031, Gaborone, Botswana; kgaboiphiwe@bvi.co.bw (K.G.); jhyera@bvi.co.bw (J.H.); efana@bvi.co.bw (E.M.F.); 2 Department of Biological Sciences and Biotechnology, School of Life Sciences, Botswana International University of Science and Technology, Private Bag 16, Palapye 10071, Botswana; tshephang.kabelo@studentmail.biust.ac.bw (T.I.K.); mp18000018@studentmail.biust.ac.bw (P.T.M.); masisik@biust.ac.bw (K.M.)

**Keywords:** foot-and-mouth disease virus (FMDV), foot-and-mouth disease (FMD), SAT serotypes, cell lines, FMD vaccine production, FMD diagnostic

## Abstract

Cell culture underpins virus isolation and virus neutralisation tests, which are both gold-standard diagnostic methods for foot-and-mouth disease (FMD). Cell culture is also crucial for the propagation of inactivated foot-and-mouth disease virus (FMDV) vaccines. Both primary cells and cell lines are utilised in FMDV isolation and propagation. Widely used cell lines for FMDV and isolation and propagation include baby hamster kidney cells (BHK-21), swine kidney cells (IB-RS-2), foetal goat tongue (ZZ-R 127), foetal porcine kidney cells (LFBKvB6), bovine kidney cells (BK), human telomerase reverse transcriptase bovine thyroid (hTERT-BTY) and porcine kidney-originating PK-15 or SK 6 cell lines. This review highlights how different receptors and molecules—integrins, heparan sulphate (HS), and the Jumonji C-domain containing Protein 6 (JMJD6)—found on the surface of different cell types contribute to differences experienced with susceptibility and sensitivity of the cells to infection with different serotypes of FMDV. This review specifically focuses on Southern African territory (SAT) serotypes, which are unique to the Southern African context and are often under-investigated in cell line development for FMDV isolation and propagation.

## 1. Introduction

Foot-and-mouth disease (FMD) is a highly contagious disease that predominantly affects cloven-hoofed animals of the order Artiodactyla, such as cattle, goats, sheep, and 70 species of wild animals [1,2]. This disease is caused by foot-and-mouth disease virus (FMDV). Transmission of this virus is usually through inhalation of droplets, contaminated feed, insemination with contaminated semen, and the use of contaminated vaccines [1]. The African buffalo (*Syncerus caffer*) is the only wildlife species confirmed to be a reservoir of the virus. The African buffalo is notorious for being a carrier of the Southern African territories (SAT) serotypes of FMDV, complicating FMD control and eradication, particularly in Southern Africa where all three SAT serotypes of FMDV circulate exclusively [3]. Complication in FMD control and eradication results from the interaction of FMDV-infected/carrier wild ungulates with FMDV-susceptible domesticated ungulates as they forage and drink on the same rangelands [4].

FMD is caused by a non-enveloped, single-stranded positive-sense RNA virus of the genus *Aphthovirus* and family Picornaviridae. The genome has two untranslated regions, one on the 5′ end and the other on the 3′ end, as well as a large single open reading frame (ORF), which post-translates into non-structural proteins (Lab, Lb, 2A, 2B, 2C, 3A, 3B, 3C, and 3D) and viral structural proteins (VPs), namely VP1, VP2, VP3, and VP4 [5]. The translation of the viral proteins begins from either of the two AUG start codons located before the leader protease (Lpro), leading to two possible isoforms: Lab and Lb. The ORF is traditionally classified into three distinct parts: P1, P2, and P3. These parts are responsible for the translation of a substantial polyprotein, which is then enzymatically cleaved into a sequence of intermediate and fully formed viral proteins [2]. The P1 region contains the genetic information for the viral structural proteins: 1A (VP4), 1B (VP2), 1C (VP3), and 1D (VP1). On the other hand, the P2 and P3 regions include the genetic information for the viral non-structural proteins: 2Apro, 2B, 2C, 3A, 3B1, 3B2, 3B3, 3C protease (3Cpro), and 3D RNA-dependent RNA polymerase (3Dpol) [6].

The nucleic acid of FMDV is enclosed by a symmetric protein shell known as the capsid, which consists of twelve pentamers each with 60 copies of capsomers, made up of the four viral structural proteins [7]. These structural proteins are also essential for viral infection and immune recognition, maintaining viral stability, facilitating cell binding to cells, and determining antigen specificity [2]. VP1 in particular is a key immunogenic site predominantly exposed on the surface of the capsid making it essential for the adsorption of FMDV and its entry into the cell [8]. The immunological distinction of VP1 gives rise to the different serotypes, namely A, Asia-1, C, O, and SAT 1, 2, 3 [9] and also accounts for the genetic variability of FMDV strains. The SAT serotypes are unique to Africa, with SAT 2 being the most predominant serotype [10,11]. SAT serotypes differ in distribution, outbreak incidence in domestic livestock, and infection rates in susceptible wildlife species [12]. Seventy-seven per cent of the global livestock in Africa, Asia, the Middle East, and limited parts of South America are at risk of infection with FMDV [9]. This prevalence distresses the cultural, social, and economic aspects of the communities affected by the virus. In sub-Saharan communities, abundant livestock is a source of social status, food, and income [12]. Africa is estimated to suffer the greatest output losses from FMD, accounting for about USD 830 million, which is approximately 17% of global yearly expenditures, making it an economic burden to the countries faced with this scourge [13].

FMD is initially recognized in susceptible animals by primary and secondary clinical signs. The primary signs usually include vesicles and erosion of cutaneous mucosae and hairless skin parts, affecting mainly the mouth and hooves [14]. Secondary signs encompass lameness, excess salivation, reduced milk production due to lesions on the mammary glands, high temperature, loss of appetite, and ultimately weight loss and anorexia [15]. In natural infections, the pharyngeal region is the main site of infection in animals. The virus initiates infection inside the pharyngeal epithelium followed by substantial replication within the pneumocytes in the lungs. One to two days after infection, the virus infiltrates the bloodstream and spreads throughout many organs and tissues to undergo further replication, inducing evident viremia [16].

### 1.1. FMD Virus Adhesion and Entry

**Figure 1:**
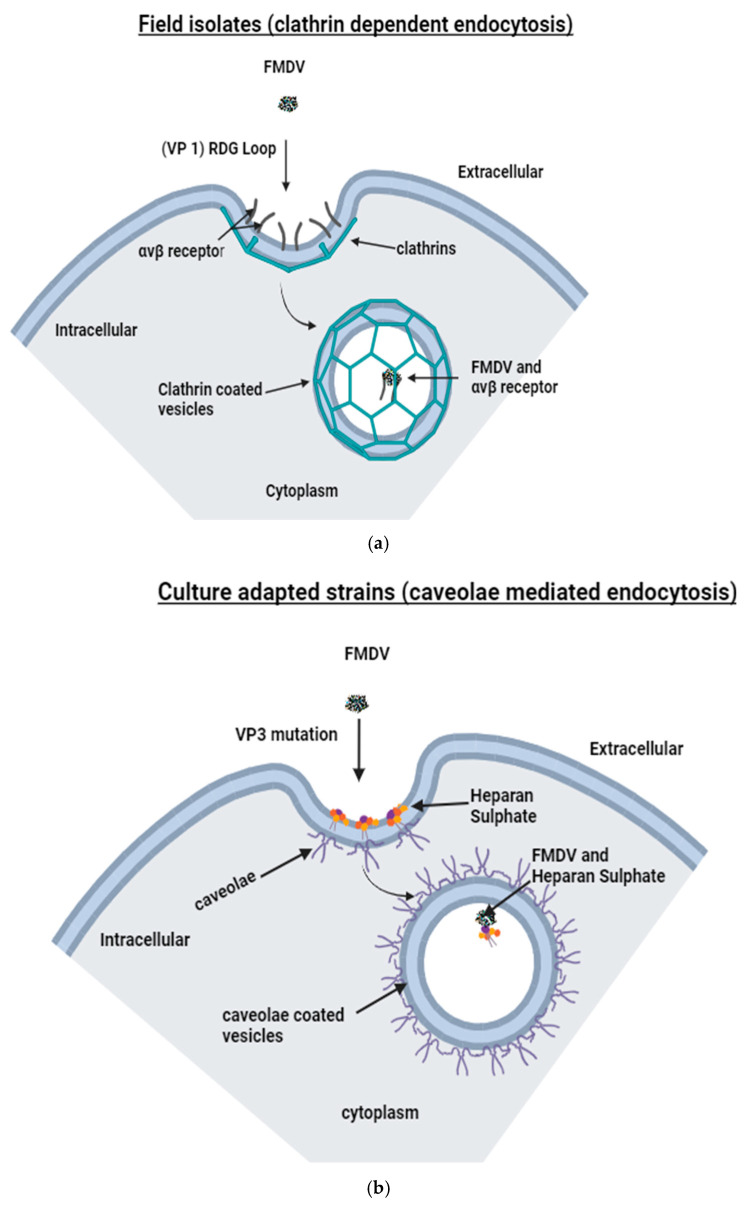

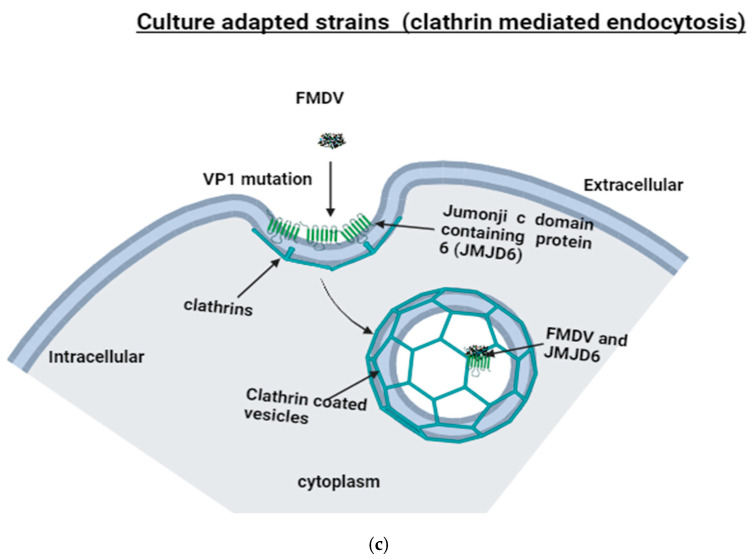
Field FMDV utilises the αVβ to enter the cells through the arginine–glycine–aspartic acid (RGD) amino acid sequence motif located within the βG-βH loop of VP1. The virus is assimilated internally by the clathrin-dependent endocytosis in the cytoplasm (**a**). In culture-adapted strains, mutant VP3 allows viral entry in the cell by caveolae-mediated endocytosis through heparan sulphate (**b**). Jumonji C-domain containing protein 6 (JMJD6) serves as a tertiary receptor to mediate clathrin-mediated endocytosis of the virus into the cell through a mutated VP1 (**c**).

The initial step of FMDV cell invasion is called cell adsorption. The adsorption process is dependent upon the presence of host cell receptors, which facilitate subsequent virus entry into host cells [17]. An example of these host cell receptors is integrins. Integrins are a family of widely dispersed receptors found on the surface of cells (Figure 1a). All members of this family consist of heterodimeric transmembrane glycoproteins that have α and β subunits, which are produced by non-covalent interactions. Integrin receptors are primarily used by the virus to commence infection [16,17]. Integrins consist of subunits that are classified as type I transmembrane proteins. These subunits consist of a sizable extracellular domain, a tiny transmembrane domain, and a cytoplasmic domain [8]. The extracellular ligand-binding domain is created by N-terminal α and β chains, creating a spherical area, which enables FMDV binding [8]. FMDV adsorption occurs by binding to any of the four members of the αV subgroup of the integrin family of cellular receptors (αVβ1, αVβ3, αVβ6, and αVβ8) via a highly conserved arginine–glycine–aspartic acid (RGD) amino acid sequence motif [18].The RGD sequence is located within the G-H loop (βG-βH) of the VP1 capsid protein of FMDV, and it has been demonstrated to create a highly stable interaction with integrin proteins found in cells [8]. The RGD sequence is the main constituent of the cell adsorption site and can bind to integrin receptors on the cell membrane to facilitate the onset of virus infection. With the exception of VP4, capsid proteins are all linked to antigenicity and have the ability to interact with the RGD-directed integrin protein subunits [8]. Among the identified receptors, αVβ3 and αVβ6 integrins are reported to be used with high efficiency by FMDV for cell entry [8]. The αVβ6 receptors are prevalent in the epithelial cells of target tissues. This observation aligns with the established knowledge that the virus has a tropism for epithelial cells throughout infection [16].

Viral adsorption through the integrins is followed by virus internalisation and the subsequent transfer of viral nucleic acid, or nucleoprotein core, to the cytosol through endocytosis [6,18,19]. FMDV infection by field viruses is facilitated by integrin-mediated mechanisms involving clathrin-dependent endocytosis [8]. In clathrin-mediated endocytosis, clathrin molecules are organised on the inner side of the plasma membrane to create a structure known as a clathrin-coated pit. The virus–receptor complex is internalised in these clathrin-coated pits, which then undergo invagination towards the cytoplasmic side and become detached from the cell membrane [20]. Acidified endocytic vesicles play a crucial role in the fast disassembly of the viral capsid protein structure. The acidic milieu facilitates the activation of the penetration processes, enabling the virus to invade the cytoplasm [8].

Although FMDV infection is primarily facilitated by the RGD motif, it is nevertheless possible for infection to occur independently of the RGD motif [8]. Cell-surface glycosaminoglycan (GAG) molecules have been linked to infections by cell culture-adapted FMDV strains. Heparan sulphate proteoglycan (HSPG) is a specific type of GAG that serves as a secondary receptor for cell culture-adapted FMDV by caveolae-mediated endocytosis [20,21]. The process is facilitated by caveolin, which are flask-shaped indentations in the plasma membrane as well as the lipid rafts, and cholesterol [20]. After FMDV enters cells by caveolae-mediated endocytosis (Figure 1b), it then moves with endosomes by taking advantage of communication within the caveolae-to-endosome transport pathway [22].

The heparan sulphate (HS) binding site is a superficial cavity on the surface of the virion, situated at the intersection of the three primary capsid proteins—VP1, VP2, and VP3 [23]. This site contains specific amino acids that form the canonical HS-binding site, which includes amino acids at positions 55 to 60 of VP3, 133 to 138 of VP2, and 195 to 197 of VP1 [24]. The HS can hold four or five sugar residues which establish connections with the three exterior capsid proteins of FMDV. The HS structure rarely changes in field viruses. In culture-adapted viruses however, the transition to binding with HS occurs due to one or two alterations in the central region of the HS-binding site, leading to an overall increase in positive charge [25]. The primary alteration is reported to occur at VP3-56, where the amino acid histidine (H) often seen in field viruses is replaced by arginine (R) in cell culture-adapted strains. The presence of the R group is crucial for the virus to bind through the HS, as it facilitates ionic interactions with two sulphate groups. HS binding has been demonstrated for various serotypes of FMDV. The receptor has been mostly studied in culture-adapted FMDV O, C and SATs [25]. It is reported, however, that cell culture-adapted FMDVs that use the HS have reduced pathogenicity [21].

In addition to integrins and HS, a mutant VP1 protein of FMDV can interact with cells through the Jumonji C-domain containing Protein 6 (JMJD6) [16,25] (Figure 1c). This is a rarely used receptor that contributes to the expansion of cell tropism by serving as a tertiary receptor for FMDV [24]. After interaction with HS, FMDV is internalised through clathrin-mediated endocytosis [26]. The JMJD6 is a phosphatidylserine receptor (PSR) that serves as a signal on the surface of apoptotic cells when the phospholipid membrane bilayer asymmetry has been disrupted [6]. The utilisation of the JMJD6 receptor by FMDV consistently follows the utilisation of the HS receptor during the cellular adaptation process of the virus. The receptor is observed to move from the cell’s nucleus to its plasma membrane during FMDV infection. JMJD6 plays a part in the induction of RNA Helicase A (RHA) arginine demethylation, as the virus enlists the assistance of host cell components to carry out its rapid replication cycle [6]. Chinese hamster ovary (CHO) cells, which do not normally show susceptibility to FMDV, become permissive to FMDV infection after a specific mutation in the VP1 protein causes the C-terminus of JMJD6 to interact with VP1. Interaction of JMJD6 with a mutant FMDV of serotype A has been shown to lead to successful viral adsorption in CHO 677 cells, which were deficient in integrins [6].

### 1.2. The Role of Cells in FMDV Diagnosis and Vaccine Production

The World Health Organization of Animal Health (WOAH) recommends two main FMDV diagnostic tests: (i) virus isolation in cell culture followed by virus typing either by antigen enzyme-linked immunosorbent assay (AgELISA) and (ii) virus genome (RNA) identification by reverse transcription polymerase chain reaction (RT-PCR) platforms [9,27]. Additional to these tests, virus neutralisation test (VNT) in cell culture, a serotype-specific serological assay, is also recommended mainly for trade purposes to monitor viral antibodies in animals. From the above-mentioned diagnostic methods, virus isolation and VNT require the use of FMDV infection-permissive cells to replicate the virus in vitro.

When samples are received from the field for laboratory FMDV diagnosis, virus isolation is the first test that is undertaken. Virus isolation is a valuable method for increasing FMDV titres, facilitating subsequent diagnostic procedures such as AgELISA and VNT [27]. Thus, virus isolation is a gold standard method in FMD diagnosis, and it involves the use of primary cells or cell lines that are highly sensitive to FMDV infection [28]. Sensitivity is a critical factor when selecting cells for virus isolation as the intent of this method is to detect and amplify trace amounts of a virus in a sample. Secondary to amplifying the virus, this test also allows for the analysis of viral morphology, as well as the characterization of viral particles. To achieve this, virus particles are allowed to infect cells and generate new virus progeny through replication. In this process of viral replication, there are changes to the morphology and biochemistry of the host cell ultimately leading to cell destruction [29]. Depending on the type of virus, these alterations can take many different forms, including syncytium development, cell rounding, or cell lysis. These observable alterations in cells, known as cytopathic effects (CPEs), allow for the detection and measurement of virus-induced cell death [29].

When field viruses are propagated and subsequently passaged or sub-cultured in cells they undergo a process referred to as adaptation. Sub-culturing FMDV has been shown to raise virus titres. Depending on the FMDV serotype strain, adaptation may require multiple/repeated passages in cell culture, a process that leads to a risk of viral antigenic variability [28,29,30]. Antigenic variation enables genetic plasticity and evolution of the field viruses in order to amplify in tissue culture [31]. The genetic plasticity and evolution frequently result in a diminished clinical phenotype in the field viruses and can alter significant physical characteristics of the viral capsid [31].

VNT is a serological test used to detect the presence of antibodies that prevent the infectivity of a virus [32]. VNT measures the titre of neutralising antibodies post-exposure or post-vaccination. This test is performed in vitro and is based on the inhibition of virus infectivity in cell culture in the presence of neutralising antibodies in serum [33]. The presence or absence of cytopathic effect or evidence of viral infection in an immune reactive technique such as VNT may be used for virus titre determination. The use of FMDV-susceptible cells is crucial since the obtained virus titres are used to determine a relationship coefficient that is essential in matching a vaccine strain and a field isolate [33].

In addition to diagnostic applications, cells are also a crucial component of vaccine production. The traditional technique for vaccine manufacture involves the propagation of viruses in cell cultures [34]. Cells selected for propagation are usually well-characterized, susceptible to FMDV, capable of producing high virus titres, and easy to scale up for large-scale production. Cells such as baby hamster kidney cells (BHK-21) can be employed to propagate large amounts of infectious and pathogenic FMDV required to generate inactivated vaccination antigens [35]. Inactivated virus vaccines have been shown to be highly effective in managing FMD. Vaccinations have also played a crucial role in eradicating the disease in developed countries. Typically, these vaccines generate neutralising antibodies and provide effective protection against similar strains of FMDV [36]. Conventional inactivated FMD vaccines, however, have a limited ability to provide long-lasting protection against a wide range of strains. This highlights the need for multiple vaccinations to maintain strong immunity, and the requirement to periodically update the vaccine to include new viral strains that are not covered by existing vaccines [36]. Consistent vaccine production requires a continuous supply of cell cultures but available cell lines such as BHK-21 present challenges in cultivating particular FMDV serotypes such as SAT in cell cultures for vaccine manufacturing since they are mostly permissive but not sensitive to the strains [35,36].

Some primary cells such as the bovine thyroid (BTY) and lamb kidney cells are used for diagnostic tests such as virus isolation and VNT [35,37]. The utility of these cells, however, has limitations, such as limited lifespan, the laborious and time-consuming process of sourcing and culturing primary cells, as well as the loss of sensitivity after cryopreservation and successive passaging [37]. The use or development of cell lines that are permissive to infection by FMDV is an underpinning solution to overcome the limitations faced when using primary cells [38]. Bovine, ovine, and porcine cell lines are used in FMD diagnostic tests and vaccine production [9]. FMDV can be propagated in cell lines such as baby hamster kidney (BHK-21,) swine kidney (IB-RS-2), foetal goat tongue (ZZ-R 127), foetal porcine kidney (LFBKαVB6), bovine kidney (BK), the human telomerase reverse transcriptase bovine thyroid (hTERT-BTY) and PK-15 and SK-6 originating from porcine kidney [37,38]. An understanding of the utility of these different cell lines in FMDV isolation and propagation is of paramount importance, especially in the context of SAT serotype viruses, which are unique to Africa.

The focus on SAT viruses is justified by their peculiarity. SAT viruses have been reported to have different infection sensitivities in different cell lines and primary cells [38,39]. For example, SAT field strains take a prolonged time to depict a cytopathic effect in some cell lines such as BHK-21 [40]. It is also important to have an understanding of the utility of different cell lines in the isolation and propagation of SAT viruses because selective pressures during the adaptation of viral strains can result in capsid modifications that affect the antigenicity and stability of the virus particle, potentially influencing receptor tropism, particle stability, and antigenicity [40]. SAT viruses are additionally important to consider in comparison to other viruses because variability in stability among and within serotypes has been reported. Jackson et al. [41] report that within each serotype, distinct strains demonstrated varying thermostabilities. Some virus strains have more stable capsids than others. Less stable strains can consequently lose infectivity when thermally compromised and pose difficulties in the isolation and propagation of the viruses [41]. This review, therefore, serves to assess the cell lines currently used for the propagation of SAT serotypes of FMDV in diagnosis and vaccine production. The review will also highlight the culture systems used for the isolation and propagation of FMDV, the abundance of FMDV permissive receptors in the cell systems, and the comparative performance of the cell lines between FMDV SAT serotypes and other serotypes.

## 2. Materials and Methods

### 2.1. Study Design

A review of studies was conducted to examine cell lines currently employed for isolating and propagating FMDV, with particular interest on SAT serotypes. The analysis identified the most effective cell lines crucial for FMD vaccine development and diagnostics.

### 2.2. Eligibility Criteria

The analysis included all original articles, experimental studies, clinical studies, and articles published in English that focused on the use of cell lines for propagating SAT serotypes of FMDV in diagnostics and vaccine production. All unpublished data, letters to the editor, reviews, and studies that lacked proper validation of their results were excluded.

### 2.3. Search Strategy

Databases searched: PubMed, Google Scholar, Ebsco, ScienceDirect and NCBI Search. Period: from September 2023 to July 2024. Search terms and keywords: various combinations of terms such as ‘Foot-and-Mouth Disease Virus’, ‘FMDV’, ‘SAT serotypes’, ‘cell lines’, ‘diagnostics’ and ‘vaccine production’ (Table 1).

Additional searches were conducted on PubMed for references cited in review articles relevant to the topic.

## 3. Results

A total of 57 original articles as well as the World Organisation for Animal Health (WOAH/O*I*E) manual were reviewed to analyse the use of cell lines for FMDV propagation, their associated receptors and the serotypes studied. The review identified eight commonly used cell lines (Table 2) and categorised them in the order of their organ origin. Integrins (αVβ1, αVβ3, αVβ6, and αVβ8), heparan sulphate and the Jumonji C-domain containing Protein 6 (JMJD6) were identified as critical molecules for cell adsorption, noting their expression in these cell lines. Additionally, the interaction of these cell lines with various FMDV serotypes was investigated, with particular attention to SAT serotypes.

## 4. FMDV Propagation Cell Lines

The available FMDV propagation cell lines have different susceptibilities to FMDV serotype strains, which is likely influenced by the viruses themselves as well as the abundance and types of receptors available on such cell lines.

### 4.1. Commonly Used Cell Lines

#### 4.1.1. Thyroid-Derived Cells

Bovine thyroid primary cells (BTY) have been identified as the most sensitive culture system in FMDV diagnostics [37,48]. BTY cells have been reported to be rich in αVβ6 receptors and are, therefore, very susceptible and permissive to a variety of FMDV serotypes [42]. However, these cells tend to lose their FMDV sensitivity when passaged or cryopreserved [37,38]. Mao et al. [37] developed the hTERT-BTY, a cell line derived by overexpressing hTERT in a PCL-neo plasmid in BTY cells. hTERT activates telomerase by over-expressing the telomerase reverse transcriptase (TERT) gene, which is essential for cellular lifespan longevity [37]. These cells were observed to maintain their adhesive capacity and all the characteristics of primary cells. To avoid the loss of sensitivity due to passaging and cryopreservation, the cell line was supplemented with thyroid stimulating hormone (TSH), bovine insulin, transferrin, and L-glutamine [37].

FMDV strains, Asia-l/HN/06, O/BY/CHA/2010, A/GD/2013, and A/WH/09 were used to assess the sensitivity of hTERT-BTY cells to FMDV infection. Only cells infected with Asia- l/HN/06, A/GD/2013, and A/WH/09 had visible CPE; however, no CPE was observed in O/BY/CHA/2010-infected cells 72 hours post-infection. Immunofluorescence, however, revealed viral distribution in the cytoplasm of cells inoculated with the serotype O strain. The total viral RNA copies of FMDV-infected hTERT-BTY cells for the four FMDV serotype strains rose sharply from six hours after infection and peaked between 48 and 60 h. It was, therefore, determined that all FMDV serotypes can infect hTERT-BTY; however, there are no reports on the performance of this cell line on SAT serotypes [37].

#### 4.1.2. Tongue Epithelial-Derived Cells

The foetal goat cell line (ZZ-R 127) developed at the Friedrich-Loeffler-Institute (FLI) in Germany is reported to be of comparative sensitivity to BTY [39]. The cell line was developed by obtaining minute fragments of mucosal tissue from the distal end of a goat tongue, which were then explanted to initiate a cell culture [39]. The cell line can be passaged continuously for at least 160 passages while remaining consistently sensitive to FMDV strains without losing morphological and growth characteristics [49]. The consistent sensitivity of this cell line through passaging is important to avoid genetic plasticity and mutation in FMDV during serial passaging. It is also reported that ZZ-R 127 can be resuscitated from storage and provide a good cell density in 1–2 days, which is a good period to respond rapidly in case of an outbreak [49]. ZZ-R 127 is reported to be responsive and sensitive to all SAT serotypes of FMDV [39]. SAT serotype viruses propagated in ZZ-R 127 gave notably higher titres as compared to results in other cells for FMDV cultivation such as BHK 21. ZZ-R 127 provided evidence of CPE in only 18–24 h [42,49,50]. The basis of the significantly increased sensitivity of the goat ZZ-R 127 cell line compared to other cell lines has not been documented in the literature. However, the expression of appropriate surface receptors on the cell surface is a crucial determinant of a cell line’s sensitivity to a certain virus [39]. The expression levels of αVβ6 are deemed necessary for FMDV tropism and these are significantly elevated in the epithelial cells of the macula densa and endometria, as well as in tongue epithelial cells and salivary glands [8]. The integrin αVβ6 is reported to be prominently present on the surface of epithelial cells in healthy tissues that are recognized as primary locations for FMDV replication and where lesions typically form; therefore, the performance of this cell line might be attributed to the high presence of αVβ6 [18,39].

#### 4.1.3. Kidney-Derived Cell Lines

Kidneys are one of the secondary organs infected by FMDV in natural infections and are thus commonly used for FMDV isolation [8]. Zabal and Fondevila [44] reported an ovine kidney (OVK) cell line that is suitable for FMD culture. The cell line was developed from the kidneys of one-day-old lambs. When infected with FMDV serotypes A, O, and C viruses, an initial, similar sensitivity to BHK-21 was observed; thereafter, a ten-fold increase in sensitivity was witnessed with successive passaging. There was no report of the performance of the OVK cell line when using SAT serotype strains, but their analysis and results indicated that the OVK cell line was suitable for FMDV-sensitive isolation and production as it portrayed superior performance to BHK 21. Wang et al. [51] reported that αvβ6 integrins, one of the major receptors that necessitate FMDV entry into the cells, are present in lamb kidney cells.

BHK-21 is an anchorage-dependent continuous cell line that was developed from day-old golden Syrian baby hamsters by Macpherson and Stocker (1962). These cells are cultured in serum-free or serum-containing media on a large-scale to propagate different strains of FMDV for vaccine production [27,33]. BHK-21 is the most commonly used culture system for FMDV, but it is reported to consistently give lower titres when infected with FMDV strains O Manisa, A Iran, A Iraq, and serotypes C, Asia 1, SAT 1, SAT 2, and SAT 3 [45]. CPE was mostly visible 48 h post-inoculation of BHK-21 with FMDV strains O Manisa, A Iran, and A Iraq and serotypes C, Asia-1, SAT 1, SAT 2, and SAT 3 [44,47].

BHK-21 is reported to exhibit β3 integrin expression and not β6 integrin; however, β3 is a poor receptor for FMDV [6,45]. A down-regulation of integrin receptors is also reported in BHK-21 with successive passaging [52]. This loss of integrins leads to the adaptation of field FMDVs and facilitates selection pressure, favouring viruses that specifically adhere to and initiate infection through cell-surface GAGs and JMJD6 [17,53]. The changes accompanying enhanced FMDV propagation in BHK-21 cells have been identified in the surface-exposed loops of the VP3 and VP1 proteins of serotype A, O, C, Asia-1, and SAT 1 viruses [19,20]. These changes in the propagation of FMDV in BHK-21 cells during culture lead to challenges when the cells are adapted to SAT viruses. Field viruses of SAT viruses were reported to have slower replication cycles, whereas virus strains that had been culture-adapted to BHK-21 exhibited faster replication cycles and a stronger capacity to cause cell death [53]. Field viruses primarily utilise αVβ6 integrin as their main receptor to commence infection, while SAT viruses adapted to cell culture utilise HS as a receptor, which explains their lower virulence when first cultured in BHK-21 cells [6,17]. Although BHK is the optimised system for the propagation of most FMDV serotype strains, it is at the expense of susceptibility to infection because BHK-21 cells have low infection-permissiveness to FMDV SAT serotypes when compared to other cell lines [38].

The bovine kidney (BK) cell line is considered sensitive to infection by FMDV [44]. To ascertain this, the cells were infected with FMD serotype strain O. CPE in BK was observed within 24 h, while in BHK-21, it was observed within 48–72 h. BK was also able to give higher viral titres than BHK-21. This was explained by the presence of αVβ6 and αVβ3 integrins found on the surface of BK. There are unfortunately no reports of the use of BK cell line on SAT strains.

The foetal porcine kidney (LFBK), a cell line of porcine origin, is reported to have the ability to propagate most FMDV serotypes. However, these cells do not have significant amounts of the αvβ6 integrin [48]. The LFBK is reported to use the cell-surface GAGs and JMJD6 in cell culture-adapted FMDV [24]. The LFBKαVβ6 cell line was developed by over-expressing bovine integrin αvβ6 on the surface of the LFBK parental cell line [6,29,30]. LFBKαVβ6 was deliberately engineered using an understanding of the cellular entry mechanisms employed by FMDV. The over-expressed integrin enhances the susceptibility of LFBKαvβ6 to FMDV. This facilitates superior utility in comparison to other FMDV cell lines such as BHK-21 and the Instituto Biologico Rim Suino pig kidney number 2 (IB-RS-2), thus making LFBKαvβ6 more effective for the isolation of SAT serotypes of FMDV from field samples [27,54]. LFBKαvβ6 has been reported to have FMDV sensitivity that is very close to that of ZZ-R 127 for the isolation of SAT 1 and SAT 2 [37]. It has also been found that LFBKαVβ6 has higher virus yields than its parental cell line LFBK [48].

Strains belonging to FMDV serotypes Asia-1, O and A were used to infect both LFBKαVβ6 and LFBK, and CPE was detectable in both the cells but the cultures of LFBKαVβ6 had a higher CPE percentage and higher titres of virus were produced [30]. One major setback of LFBKαVβ6, however, is its contamination with the non-cytopathic bovine viral diarrhoea virus (BVDV), a virus that is usually found in bovine serum. The use of these cells is, therefore, hindered by the presence of BVDV, which can potentially interfere with diagnostics, virus growth, and more importantly, affect vaccine quality and productivity [27,31]. Gray et al. [54] and LaRocco et al. [28] have, however, been able to clean BVDV from the cell line, rendering it BVDV-free and safe for in vitro use.

The Instituto Biologico Rim Suino pig kidney number 2 (IB-RS-2) cell line was developed from female pig kidneys by Dr de Castro in Brazil [55]. The cell line was derived from a clone at the 154th passage, which was found to be very susceptible to FMDV [55]. IB-RS-2 cells typically arrange themselves into a monolayer that resembles epithelial tissue. When infected by FMDV, these cells undergo a process where they become rounded, detach from one another, and ultimately experience cell lysis [44]. IBRS-2 cells mostly express αvβ8 as the primary receptor for FMDV, rather than αvβ6, but IBRS-2 cells have exhibited susceptibility to all SAT serotypes with a viral isolation rate of 69% [56]. IB-RS-2 cells are reported to have reduced sensitivity to FMDV in comparison to primary bovine thyroid cells, possibly due to the limited expression of αVβ6 integrin on their cell surface [30]. When infected with SAT 1, SAT 2, and SAT 3 viruses, IB-R-2 resulted in lower titres when compared to highly sensitive cell lines such as ZZ R 127 and LFBKαvβ6 and also displayed a limited sensitivity to field strains of A, O and C [30,48]. However, these cells are easily permissive to A and C serotype viruses of porcine origin. Unfortunately, IB-RS-2 is reported to have a declining sensitivity to FMDV infection, with subsequent passaging leading to a necessity to revive a new batch when used for FMDV isolation and other serological tests. This is also reported by Zabal and Fondevila [43], who state that the usage of distinctive clones, passages, and manipulation methods to propagate viruses on the IB-RS-2 cell line can result in a 2-log difference in sensitivity in inter-laboratory testing.

Porcine kidney 15 (PK-15) and swine kidney 6 (SK-6) [39] are porcine kidney-derived cell lines. These cell lines are commonly used for FMDV research and vaccine production [20]. Du et al. [57] demonstrated a distribution of the αv, β3, and β6 when conducting expression analysis of porcine integrins, making porcine kidneys suitable candidates for FMDV replication. The SK-6/PK-15 cells were observed to exhibit CPE upon infection with the FMDV serotype O strain. The morphological characteristics seen in SK-6 cells infected with FMDV were comparable to those of PK-15 cells [58]. Though these cell lines are reported to be less susceptible to FMDV, they are readily accessible and available for use in large scale propagation FMDV [44]. Porcine cell lines, however, tend to be more susceptible to FMDV strains that are of porcine origin compared to those that are found in other vulnerable species [37,49].

Several cell lines are currently used as substrates for the isolation and propagation of FMDV. Most of these cell lines are kidney-derived cell lines, all with varying degrees of sensitivity to infection with the FMDV. The kidney, as a secondary organ in FMDV infections, works well for FMDV propagation. The kidneys also have a distribution of αvβ3, αvβ6, and αvβ8, which are important in the tropism of FMDV in cells [56]. Cell susceptibility to infection depends on the virus’s ability to bind to cell membrane receptors, and this determines how sensitive cell lines are to particular viruses [31]. Titration of the same virus has been observed to yield different viral titres in different cell lines [39]. Cell systems with poor distribution of appropriate receptors exhibit deficiencies with susceptibility to infection and consequently result in low virus yields. The primary feature that sets cell systems apart is the ability to isolate the virus particles or more specifically the presence of appropriate receptors that can mediate the adsorption of the virus in these cells [28,56]. The stability of the VP1 capsid protein of FMDV and cellular integrin proteins in forming a combination with the RGD sequence of the G-H loop is important in viral infection [8]. A helical shape near the RGD sequence is necessary for the stability of binding to αVβ6, as shown by the substitution of certain amino acids. The critical locations for this stable connection are the leucine residues at the RGD + 1 and RGD + 4 sites. This complex’s stability influences the likelihood of viral adsorption and internalisation, which in turn affects the susceptibility of the cells to FMDV [8]. The amino acid substitutions in the VP1 region are also an influential factor in the susceptibility of the virus to different cells. Jamal and Belsham [27] notably outlined that the RGDL receptor-binding motif of serotype Asia-1 viruses was more varied when compared to FMDV serotypes O and A. Variations in the amino acid residues of FMDV capsid proteins, which produce viral particles, may have an impact on the biological properties of viruses as well as their capacity to bind to cell receptors [8].

The analysis of some cell lines reviewed in this paper such as the hTERT-BTY, BK, and ovine cell lines did not include SAT strains when characterising their cell lines. The reports mainly cover serotypes such as Asia-1, O, and A. The interconnectedness of the trade of livestock and livestock products, however, makes it important to be inclusive of all serotypes when researching FMDV. Inclusivity is important for making decisions regarding FMD containment during outbreaks, diagnostics and vaccine production applications.

## 5. Conclusions

The use of cells in FMDV propagation is crucial given the economic importance of FMD. Based on the literature, ZZ-R 127 and LFBKαVβ6 cell lines seem to be the most reliable culture systems for FMDV propagation. The hTERT-BTY is also reported to be a good culture system, but there are no performance assessments of their response to infection with FMDV SAT serotypes. This highlights the gap in research on the sensitivity of different cell lines to infection with different FMDV serotypes. The inclusion of SAT strains in research will provide comprehensive reference information necessary for choosing the right cells for FMDV identification, characterization, propagation and vaccine matching. Additionally, more work needs to be undertaken to develop new cell lines and/or improve existing ones to better suit SAT serotypes and minimise their tendency to adapt and utilise alternate receptors.

There is a noted correlation between the origin of a cell line and its sensitivity to FMDV. Cell lines that originate from primary organs of FMDV infection such as the tongue and the thyroid tend to be more sensitive to FMDV infection. The cells from primary organs are most used in the virus isolation of field viruses while the secondary organs are used in large-scale propagation. Cell lines that are most effective for virus isolation and propagation primarily express αVβ6 as compared to other integrin receptors. As such, more research can be directed towards developing cell lines from primary organs. Further work can also be undertaken to develop holistically FMDV-sensitive cells. This would be useful as it will offer a universal platform for FMDV isolation and propagation. These advancements would not only assist effective FMD control but will ensure a more consolidated response to FMDV outbreaks across different regions globally.

## Figures and Tables

**Table 1:** Keywords and search terms.

Keywords	Combinations
Foot-and-Mouth Disease Virus	AND cell lines
FMDV	AND SAT serotypes
FMDV	AND receptors
Cell lines	AND diagnostics
Diagnostics	AND FMDV
Propagation	AND FMDV

**Table 2:** Summary of the findings on the different FMDV propagation cell lines.

Cell Line	Origin	FMDV Serotypes Propagated in the Cells	Receptors	References
Bovine thyroid cells—telomerase reverse transcriptase-(hTERT-BTY).	Bovine thyroid	A, Asia-1, O	αVβ6	[37,42]
Foetal goat cell line (ZZ-R 127)	Goat tongue	A, Asia-1, C, O, SAT 1, SAT 2, SAT 3.	presumably αVβ6	[39,42]
Ovine Kidney cell line (OVK)	Ovine kidneys	A, C, O	αv-	[43]
Bovine kidney (BK)	Bovine kidneys	O	αVβ6, αVβ3	[44]
Baby hamster kidney (BHK-21)	Hamster kidneys	A, Asia-1, C, O, SAT 1, SAT 2, SAT 3.	αVβ3, heparan sulphate, JMJD6	[6,17,21,31,41,42,45]
LFBKαvβ6 LFBK	Porcine kidneys	A, Asia-1, O, SAT 1, SAT 2, SAT 3.	Overexpressed αVβ6, heparan sulphate, JMJD6	[18,21,29,30]
Instituto Biologico Rim Suino pig kidney number 2 (IB-RS-2)	Porcine kidneys	A, C, O, SAT 1, SAT 2, SAT 3.	αVβ8, αVβ6	[29,42,43,44,46]
Porcine kidney 15 (PK-15) or swine kidney 6 (SK-6)	Porcine kidneys	O	αVβ8, αVβ6, αVβ3	[37,39,46,47]

## Data Availability

Data sharing is not applicable to this article as no datasets were generated or analysed during the current study.
